# Digital Twin-Based Improved YOLOv8 Algorithm for Micro-Defect Detection of Labyrinth Drip Emitters in High-Speed Agricultural Production Lines

**DOI:** 10.3390/s26072220

**Published:** 2026-04-03

**Authors:** Renzhong Niu, Zhangliang Wei, Peilin Jin, Qi Zhang, Zhigang Li

**Affiliations:** 1 College of Mechanical and Electrical Engineering, Shihezi University, Shihezi 832000, China; nrz1994@stu.shzu.edu.cn (R.N.); zhangqi026@stu.shzu.edu.cn (Q.Z.); 2 College of Information Science and Technology, Shihezi University, Shihezi 832000, China; wzl_shzu@shzu.edu.cn (Z.W.); jinpeilin@stu.shzu.edu.cn (P.J.)

**Keywords:** drip irrigation, labyrinth emitter, defect detection, improved YOLOv8, digital twin, DySnakeConv

## Abstract

In water-scarce regions such as Xinjiang, China, agricultural development is constrained not only by limited water resources but also by a strong reliance on water-saving irrigation technologies. Drip irrigation is a key measure for improving irrigation efficiency and promoting the sustainable development of water-saving agriculture. However, defects arising during the manufacture of labyrinth Drip emitters—the core components of drip irrigation systems—can undermine system reliability, leading to channel blockage and non-uniform irrigation. To tackle this issue, a defect detection approach is developed by integrating Digital Twin technology with an enhanced YOLOv8 model for online inspection of labyrinth Drip emitters on drip irrigation tape production lines. In parallel, a self-built dataset covering six defect categories is established. Supported by the DT framework, the standard YOLOv8 network is refined to strengthen its capability in identifying complex micro-defects. Specifically, DySnakeConv is introduced to better represent the curved and slender characteristics of labyrinth channels; DySample is incorporated to improve the reconstruction and representation of fine-grained details; an Efficient Multi-Scale Attention module is adopted to capture richer contextual information while suppressing background noise; and Inner-SIoU is applied to optimize the bounding-box regression process. Experimental results show that the model achieves 89.6% precision, 90.9% recall, and 93.9% mAP50. Compared with the baseline YOLOv8, precision, recall, and mAP50 are improved by 7.3, 3.9, and 3.3 percentage points, respectively. Under the same training conditions, the proposed model outperforms YOLOv10 and YOLOv11 in accuracy-related metrics. Specifically, compared with YOLOv11, precision, recall, and mAP50 are improved by 4.8, 5.0, and 2.6 percentage points, respectively; compared with YOLOv10, they are improved by 10.0, 7.7, and 7.3 percentage points, respectively. Meanwhile, the model maintains a lightweight size of 3.7 M parameters and a real-time inference speed of 150.2 FPS, demonstrating a favorable accuracy–efficiency trade-off. By extending manufacturing-level quality control to agricultural applications, the approach helps ensure uniform irrigation and improve water-use efficiency, providing practical technical support for precision agriculture in arid regions.

## 1. Introduction

Drip irrigation is a key technology in modern agriculture for improving water-use efficiency. The labyrinth emitter, as a core component, dissipates pressure and regulates flow through its tortuous channels, enabling stable water delivery near crop roots. Its performance directly affects irrigation reliability and quality [1,2]. Manufacturing quality, therefore, determines service life, water distribution uniformity, and machining precision. During high-temperature injection molding, mold wear, material impurities, and process fluctuations can cause burrs, deformation, and partial blockage. If these defects are sealed inside the drip tape, they may reduce emitter function, trigger irrigation failure, and cause economic loss [3].

Inspecting labyrinth drip emitters on industrial production lines remains difficult. Vibratory sorting disks can remove only simple dimensional defects, and their reliability cannot satisfy overall quality requirements [4]. Traditional image-processing methods improve efficiency, but they rely heavily on hand-crafted features and are sensitive to lighting variation, surface glare, background noise, and motion blur in factory environments [5]. In practice, inspection is difficult because the defects are very small and the patch structure is highly complex. The dense, winding labyrinth channels make it easy to confuse real defects with normal geometric shapes. Data imbalance is another challenge. In high-quality production lines, qualified products constitute the vast majority. The high acquisition cost and scarcity of critical defect samples easily lead to overfitting or missed detections during model training [6].

With the rapid development of machine learning and deep learning, end-to-end representation learning methods, especially convolutional neural networks, have shown strong generalization ability in industrial vision. Among them, the YOLO series has become a mainstream architecture for real-time industrial detection because of its balance between accuracy and speed [7]. In recent years, numerous scholars have attempted to introduce YOLO into the inspection of precision components. For example, in the defect detection of fasteners, bearing surfaces, and electronic components, significant achievements have been made by improving the network structures [8]. Deep learning has also provided effective support for plastic product defect detection. However, the micro-structural characteristics of labyrinth drip emitters require stronger fine-grained feature extraction. Direct use of the standard YOLOv8 model may still result in a weak perception of tiny defects and limited feature aggregation efficiency.

In recent years, deep learning has become a core technology for plastic product defect detection in industrial quality inspection. Plastic products are prone to bubbles, scratches, and short shots during injection molding and extrusion, while manual inspection suffers from low efficiency and inconsistent standards. Zhu et al. proposed a CNN-based model for plastic surface defect detection and improved the capture of fine flaws through multi-scale feature fusion, achieving high precision and recal [9]. D. Ideta et al. developed an automatic plastic bottle recognition system based on a deep residual network and addressed feature extraction difficulties caused by highly reflective surfaces in conveyor-belt environments [10]. Long, F et al. combined autoencoder reconstruction with CNN to achieve high-precision classification of industrial waste, including plastics [11]. Jia, Z et al. integrated hyperspectral imaging with transfer-learning Inception-ResNet-v4 for rapid and non-destructive classification of 45 automobile lampshade samples into six categories, with accuracy reaching 97.3% [12]. F For assembly lines with strict real-time requirements, Kang et al. introduced lightweight models such as MobileNet to reduce computational latency while maintaining classification accuracy [13]. Martínez, SS et al. further compared several advanced deep learning architectures for plastic classification and provided a useful reference for model selection in industry [14].

Deep learning-based object detection has advanced rapidly in industrial defect detection, although major challenges remain, as reviewed by Ma et al. [15]. For multi-scenario industrial inspection, He et al. proposed MSI Detector, a unified defect detection method based on a visual foundation model [16], To improve model efficiency, Wu et al. developed a lightweight end-to-end deep learning framework and applied it to blade defect detection [17], For image degradation in complex environments, Zhao et al. combined dehazing with an improved YOLOv8 model, which improved defect detection under water-fog interference [18].

This study proposes an improved scheme based on YOLOv8 to address three practical requirements in labyrinth emitter defect detection, namely fine-detail preservation, slender curved-structure modeling, and lightweight real-time deployment. The model is optimized from three aspects, including feature representation, scale perception, and bounding-box regression, by integrating DySample, EMA, DySnakeConv, and Inner-SIoU. Specifically, DySample [19] is used to replace the traditional interpolation-based upsampling operation, because it can recover finer-grained structural details through dynamic sampling with relatively low computational cost, making it more suitable for preserving tiny defect cues within narrow labyrinth channels. EMA [20] is inserted at the end of the backbone to enhance multi-scale channel-spatial interaction before feature fusion. This module is selected mainly because it can achieve more effective multi-scale feature enhancement at relatively low complexity, making it more suitable for suppressing repetitive texture interference on the patch surface and improving defect discrimination under industrial imaging conditions. DySnakeConv [21] is introduced because the labyrinth channels exhibit typical slender and curved geometric characteristics, and its adaptive kernel morphology is more directly aligned with such curved structures than conventional square kernels, thereby strengthening the modeling capability for subtle defects distributed along the channels. In addition, Inner-SIoU [22,23,24] is adopted to replace the original CIoU loss in order to improve localization optimization for small-scale and irregular defects. Through the joint constraints of angle, distance, and shape, together with the auxiliary bounding-box mechanism, it helps improve regression convergence and localization stability. Overall, the introduction of these modules constitutes a combined optimization strategy tailored to the current industrial scenario and task characteristics, with the aim of improving the applicability and engineering practicality of labyrinth emitter defect detection.

Following the logic of comparative machine-learning evaluation, the contribution of this work is assessed not only by accuracy-related metrics but also by deployment-related efficiency indicators [25].

This study constructs a detection framework for labyrinth emitter inspection that integrates geometry-aware representation with deployment-oriented design. Specifically, according to the structural characteristics and imaging properties of labyrinth Drip emitters, DySample, EMA, DySnakeConv, and Inner-SIoU are collaboratively configured in a task-driven manner to improve fine-detail preservation, curved-topology adaptation, background interference suppression, and the stability of tiny-defect localization. In addition, a digital twin-based offline validation environment is introduced to support inspection-station layout verification and pre-deployment feasibility analysis. Therefore, the contribution of this work mainly lies in its methodological integration for a specific industrial scenario and its engineering-oriented adaptation and optimization.

## 2. Materials and Methods

### 2.1. Manufacturing Process in the Drip Irrigation Production Workshop

The production of drip irrigation products depends on a coordinated sequence of process steps, and product quality can be ensured only when the key operations are closely connected. In the workshop, the overall workflow mainly includes raw-material thermoplastic forming, defect inspection, and production-line assembly. During manufacturing, the products further undergo precision punching, visual inspection, fixed-length cutting, and final packaging. A systematic analysis of this process chain helps identify the specific stages at which labyrinth emitter (patch) defects are generated and clarifies how such early defects may develop into functional failures in later stages.

**Figure 1:**
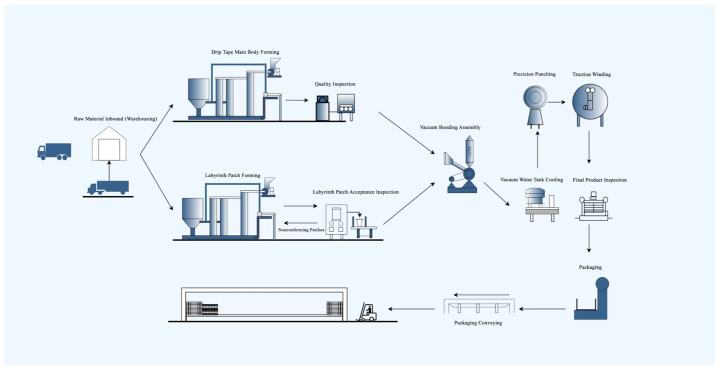
Drip Irrigation Product Manufacturing Workshop Process.

As shown in Figure 1, the process mainly includes raw-material inspection, temperature-controlled plasticization, patch molding, tape extrusion, vacuum-assisted assembly of the labyrinth emitter and tape body, cooling and drying, visual positioning, precision punching, and final winding, cutting, and packaging.

**Figure 2:**
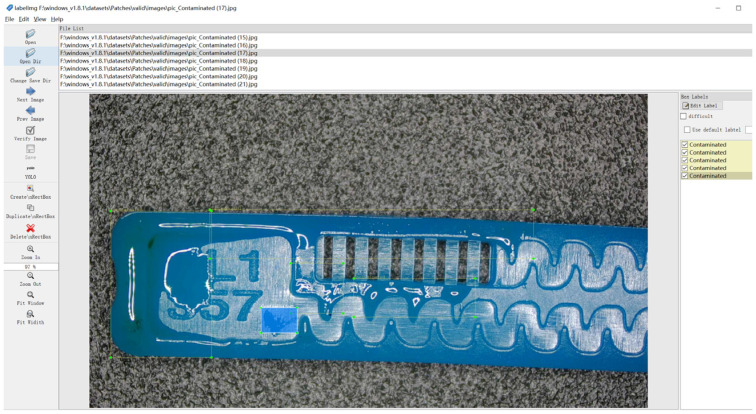
Annotation example of emitter defect samples using LabelImg.

To ensure labeling reliability, all defect samples were uniformly annotated using LabelImg 1.8.1, as shown in Figure 2. The labels were defined according to the appearance and structural quality requirements of the product, together with the production and quality-control criteria of the actual production line. Categories with ambiguous boundaries were further reviewed by quality-control personnel and domain experts, and samples without consistent agreement were excluded.

From a quality-control perspective, emitter inspection is a critical step on the production floor. Several typical defects can arise during the thermoplastic forming of the labyrinth emitter (Table 1). Once a defective patch is sealed into the drip tape wall, the internal structural damage is usually irreversible. Later steps, such as cooling, drying, and punching, cannot repair these embedded defects. Instead, the defects remain trapped inside the tape. In the field, they may lead to uneven water and fertilizer distribution, unstable outflow, early clogging, leakage, and a shorter service life.

**Figure 3:**
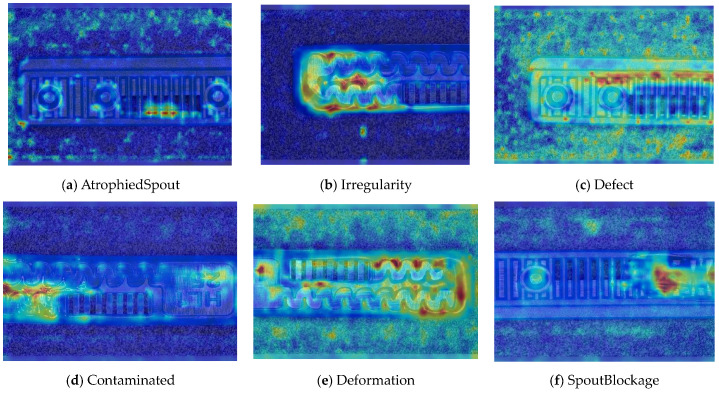
Defect Heatmap of Labyrinth emitter.

To ensure engineering consistency in the definition of defect categories, this paper classifies the typical defects of the labyrinth emitter into six categories: atrophied spout, irregularity, defect, contamination, deformation, and spout blockage. In the automated production and quality control stages of precision drip irrigation nozzles, to ensure the structural integrity of the labyrinth emitter, the stability of hydraulic performance, and the service life of the products, the visual inspection system focuses on identifying and classifying these six major categories of typical defects. First, for the AtrophiedSpout category, the system mainly detects unfilled structures and incompleteness caused by material shortage during injection molding; second, the Irregularity form encompasses flashes caused by loose mold clamping and burrs remaining at the flow channel openings, aiming to eliminate excess appendages on the edges; within the Defect category, the focus is on identifying micro-cracks induced by stress or external forces to prevent fracture failure; the Contaminated category integrates lubricating oil pollution and scorch marks generated by uneven heating to control surface cleanliness; furthermore, the system needs to identify geometric Deformation caused by uneven cooling, as well as SpoutBlockage triggered by residual impurities. Achieving the precise detection of the aforementioned six types of defects is the key to ensuring the water application uniformity and long-term operational reliability of the emitters. Typical data is shown in Figure 3 below.

**Figure 4:**
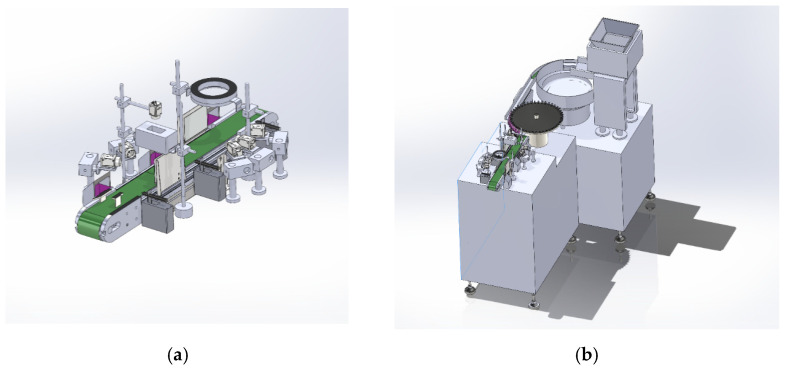

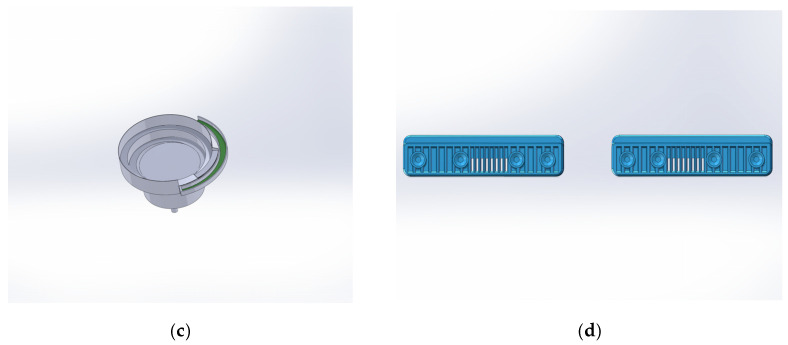
Digital twin of key processes: (**a**) Visual inspection of labyrinth emitter; (**b**) Discharge inspection port; (**c**) Vibratory Screening Tray; (**d**) Labyrinth emitter.

Furthermore, patch inspection directly supports the “precision punching” process. Since the holes must be aligned with the center of the patch within strict tolerances, any positional deviation, deformation, or localized damage to the patch can result in eccentric punching, further impairing hydraulic performance and elevating the risk of scrap. Therefore, a stable visual inspection and positioning module serves not only for defect screening but also for stabilizing the entire production line. This paper further constructs a digital twin of the production line in an offline simulation environment as shown in Figure 4, for the system-level demonstration and verification of the inspection station layout and data flow organization.

**Figure 5:**
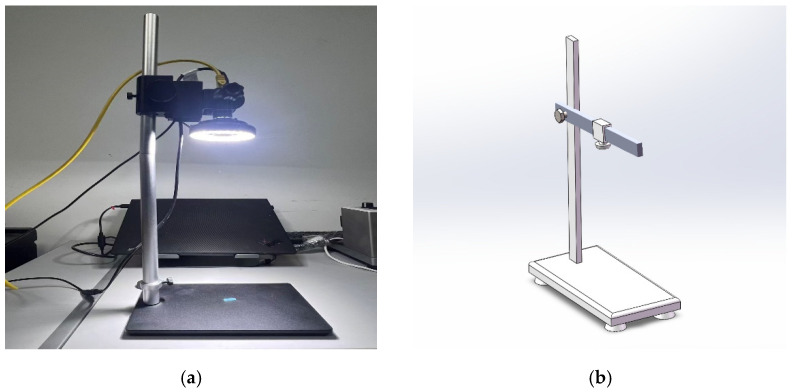
Testbed simulation: (**a**) Laboratory data acquisition workbench; (**b**) Simulation testbed.

In this study, a custom-built visual acquisition workbench was established in the laboratory, utilizing a Hikrobot Gigabit Ethernet industrial area scan camera MV-CA060-11GC (6 megapixels hikvision, Hangzhou, China) for image acquisition. This camera employs a global shutter CMOS sensor (IMX178 hikvision, Hangzhou, China) with a resolution of 3072 × 2048. The acquisition system’s supporting equipment includes a 12 V power adapter, a Gigabit Ethernet cable, an adjustable bracket and pan-tilt head, a ring light source and light controller, and a checkerboard calibration board (used for camera calibration and pixel-to-physical scale conversion). During the acquisition process, the camera was mounted on the front end of the bracket via the adjustable pan-tilt head, with the lens’s optical axis kept vertically downward (top-down view), capturing images of the Labyrinth Emitter samples placed on the workbench under uniform illumination conditions. The object distance was set to 0.8 m, which corresponds to a physical acquisition range of approximately 0.8 m × 0.5 m covered by a single image (as shown in Figure 5). A total of 3000 original images were collected, covering six defect categories, with 500 images for each category. Using stratified random sampling, the self-constructed dataset was strictly divided into training, validation, and test sets at a ratio of 8:1:1 to ensure balanced class distribution across all subsets. The test set remained unseen during both training and hyperparameter tuning and was used only for final inference and performance evaluation. It should be noted that the raw acquired images were not subjected to any additional traditional image preprocessing operations before model training, such as denoising, histogram equalization, contrast enhancement, or defect-region enhancement. In this study, the original imaging characteristics were largely preserved, and the images were processed only according to the standard input requirements of YOLOv8, including image resizing and normalization, while the default data augmentation strategy of the YOLOv8 framework was adopted during training. Therefore, the detection results reported in this paper are based on raw acquired images processed through the above standard input pipeline, rather than on manually enhanced or specially preprocessed inputs.

### 2.2. Improved YOLOv8-Based Detection Method

**Figure 6:**
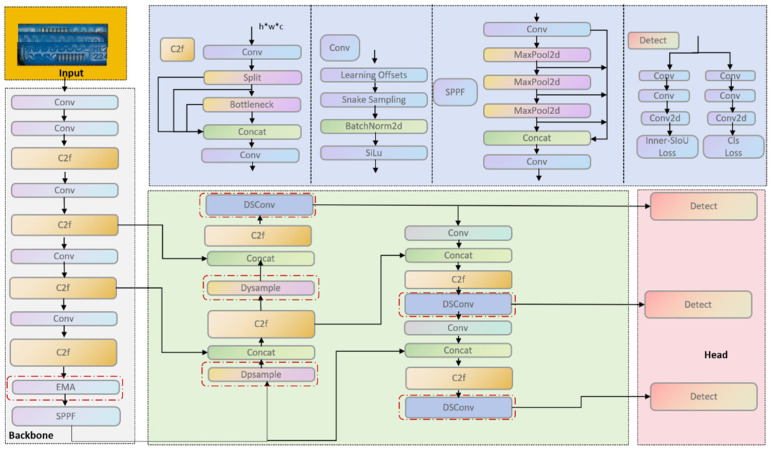
Overall architecture of the improved YOLOv8 network. Regions highlighted by red dashed boxes denote where the enhanced modules are integrated.

This method improves the YOLOv8 model in the target detection algorithm. The proposed approach enhances the ability to extract image defect features during the training process, improves the detection rate, and reduces false detections and missed detections. As a result, the capability of identifying various defects in the labyrinth emitter produced in the drip irrigation production workshop is significantly improved. The structure of the improved network model is shown in Figure 6.

For reproducibility, the implementation details of the improved architecture are summarized as follows. DySample is used to replace the original upsampling operation in the neck feature-fusion pathway. EMA is inserted at the end of the backbone to enhance multi-scale channel–spatial interaction before feature fusion. DySnakeConv is introduced into the feature fusion and propagation stages to strengthen the modeling of slender and curved channel structures. In addition, the original CIoU loss used for bounding-box regression is replaced by Inner-SIoU, while the remaining detection pipeline of YOLOv8 is kept unchanged unless otherwise stated. These modifications are implemented on the basis of the original YOLOv8 framework without introducing an entirely new detector architecture.

To address the challenges of identifying micro-defects in labyrinth Drip emitters within complex production environments, this study proposes an optimized and enhanced YOLOv8 framework, focusing on strengthening the model’s perception of subtle features and its high-fidelity reconstruction capability. As illustrated in Figure 6. By adding the DySample dynamic upsampling operator and the Efficient Multi-ScaleAttention module, the model becomes more discriminative in complex backgrounds with repetitive textures. Overall performance is better than that of standard network designs. DySample replaces the conventional static upsampling layer. It uses learned spatial offsets to perform content-adaptive resampling. This reduces feature spreading and edge blurring that often occur with interpolation-based upsampling. It also restores fine structures, such as flow-channel boundaries, with low computational cost. Meanwhile, EMA is placed at the end of the backbone. It employs parallel sub-networks to capture multi-scale and cross-dimensional interactions. Compared with common attention designs that may lose information during dimensionality reduction, EMA preserves feature integrity more effectively. As a result, the model can still build stable global semantic relations even under noisy industrial imaging conditions. Taken together, these changes lower false positives and missed detections. They provide more reliable technical support for surface defect inspection in drip-irrigation production workshops. They also yield richer multi-scale representations, which help distinguish key cues among different patch defects. In addition, Dynamic Snake Convolution is introduced to match the elongated and highly curved geometry of irrigation channels. This module uses a cumulative offset mechanism. It allows convolution kernels to deform and adapt to structural changes. The kernel can follow the snake-like channel layout more closely. This improves feature extraction for slender and irregular defects. It also enhances detection performance when strong geometric constraints are present. The original CIoU bounding box loss function is optimized to Inner-SIoU. By introducing an Auxiliary Bounding Box and an angle cost function, Inner-SIoU improves the angle alignment problem during the regression process. Furthermore, by adjusting the scale of the auxiliary box, it accelerates the regression convergence for extremely small targets and targets with high aspect ratios, thereby improving localization accuracy and regression stability.

#### 2.2.1. YOLOv8 Network

YOLO (You Only Look Once) is a class of efficiency-oriented single-stage object detection methods, widely used in practical applications due to its end-to-end fast inference characteristics. This framework unifies the detection process into regression-based prediction: the model no longer relies on the step-by-step screening of candidate regions, but directly learns bounding box parameters and class probabilities on divided grid cells, thereby accomplishing localization and classification in a single forward pass [26]. In YOLOv8, the network pipeline can be summarized into four stages: input preprocessing, feature extraction, feature fusion, and prediction output. In terms of feature extraction, the backbone network of this model still adopts the ELAN method used in YOLOv7. Compared with YOLOv5, the C2f module is introduced to replace the C3 module, enabling features to be transmitted through more abundant gradient pathways while balancing receptive field expansion and computational cost control. Combined with the SPPF structure, it further enhances the multi-scale context aggregation capability to accommodate the detection requirements of targets with varying sizes. Subsequently, the Neck accomplishes the synergistic fusion of semantic and spatial information through cross-scale upsampling and multi-layer feature combination, thereby improving the quality of feature representation and localization consistency. The prediction end adopts a decoupled head structure, separating the classification branch from the regression branch to reduce inter-task interference, and shifts from an anchor-based mechanism to an anchor-free design. This reduces sensitivity to predefined anchor box sizes, thereby simplifying model configuration and enhancing cross-scenario generalization performance.

#### 2.2.2. Dynamic Upsampling

**Figure 7:**
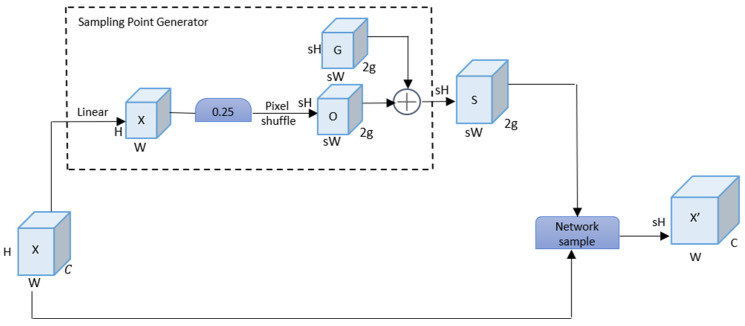
Basic structure of Dysample.

YOLOv8n utilizes nearest-neighbor interpolation for upsampling to adjust the size of the input feature map. However, this method directly copies the nearest pixel values, which leads to the loss of detail information in the image. In particular, the complex textures or detailed features of the Labyrinth emitter are difficult to preserve effectively. Therefore, DySample is introduced to replace the nearest-neighbor interpolation method. DySample is a point-sampling-based upsampling method [27], which assigns different weights to the pixel neighborhood around each sampling point during upsampling based on the feature information of different regions in the sample image. In this way, DySample can restore image details more accurately. Figure 7 shows the basic structure of DySample.

Here, s is the upsampling scale factor, and 2g represents the (x,y) coordinates. Given a feature map of size C×H×W, a linear layer with input channels C and output channels $2s^{2}$ is used to offset the position of the input feature map. To avoid the overlapping of local $s^{2}$ sampling positions, a static scope factor is introduced, that is, the offset is multiplied by 0.25 to achieve local sampling position constraints, generating an offset O of size $2s^{2}\times H\times W$. Then, through Pixel shuffle, it is reshaped into 2×sH×sW, and the sampling set is jointly composed of the original sampling grid G and the offset O. Finally, the positions in S are used to sample the interpolation χ into the feature map vector $\chi^{\prime }$. The formula is:

(1) $$O=0.25Linear\left(\chi \right)$$

(2) S=G+O

(3) $$\chi^{\prime }={grid}_{-}sample\left(\chi ,S\right)$$

Furthermore, the offsets of sampling points may overlap, thereby leading to detection errors. A dynamic scope factor is generated through the linear projection of input features, which is used to adjust the offset O to reduce overlap. Linear layers are separately employed to perform the offsets on χ; one side is constrained using a static scope of 0.5, while the other side remains unconstrained, ultimately yielding the final offset O.

(4) $$O={0.5Sigmoid\left({Linear}_{1}\left(\chi \right)\right)Linear}_{2}\left(\chi \right)$$

The ultra-lightweight dynamic upsampling module, DySample (as shown in Figure 6), is adopted to replace the native Upsample module. Through a dynamic point sampling mechanism, this improvement strengthens the model’s capability to restore the structural details of the Labyrinth emitter while reducing computational overhead. Under the premise of decreasing network complexity, it significantly enhances the model’s capture efficiency for minute and edge-sensitive defects, enabling the network to more acutely perceive semantic changes within the labyrinth channels.

The original model employs Nearest Neighbor Interpolation for upsampling. However, this method expands the image merely by directly copying the nearest pixel values, which is highly prone to causing the loss of high-frequency detail information in the image. Especially for labyrinth emitter defect detection, the labyrinth-channel textures on the patch surface are highly complex. Micro-defects such as tiny blockages and edge breakages are often hard to notice. When traditional interpolation-based upsampling is used, these critical details are not well preserved. This can reduce detection accuracy. Addressing this limitation, this paper introduces the DySample module to replace the original nearest neighbor interpolation method. DySample is a lightweight upsampling method based on point sampling [28]. Unlike static interpolation, it can adaptively assign weights to the pixel neighborhood around each sampling point during the upsampling process based on the semantic features of different regions in the sample image. In this way, DySample can more accurately reconstruct the structural texture of the Labyrinth emitter and effectively restore the detailed information of minute defects.

#### 2.2.3. Efficient Multi-Scale Attention Network

In complex industrial scenarios, defects are typically characterized by large-scale variations, repetitive textures, and strong background interference. Traditional convolutional networks tend to be biased towards locally salient regions, leading to insufficient global information fusion, which in turn causes false positives or false negatives. To enhance the multi-scale representation capability of features at the end of the backbone network and to suppress the influence of illumination fluctuations and background noise, this paper introduces the EMA module [29] at the end of the backbone network. It achieves adaptive enhancement of key defect regions through group modeling + spatial directional attention + joint channel/spatial recalibration.

The Efficient Multi-scale Attention mechanism adopts a grouping strategy to achieve joint channel-spatial attention modeling. Given the input feature $X\in R^{B\times C\times H\times W}$, EMA divides it into G groups (default G = 32) along the channel dimension, with the number of channels in each group being $C^{\prime }=C/G$, ultimately obtaining $X_{g}\in R^{(B\cdot G)\times C^{\prime }\times H\times W}$. EMA aggregates and recalibrates the group features through three parallel branches: two of the branches are located within the 1 × 1 path, and their processing flow is similar to the CA (Coordinate Attention) mechanism—performing one-dimensional average pooling along the horizontal and vertical directions respectively to encode cross-channel dependencies with positional information. This is followed by feature concatenation and the generation of responses through a 1 × 1 convolution; since grouping has already been performed, this process does not require additional channel dimensionality reduction.

Subsequently, the convolutional output is split and passed through a Sigmoid function, respectively, to obtain two attention maps, which are then fused via element-wise multiplication to form the intra-group channel recalibration weights, thereby highlighting the edge and texture clues of the patch defects. The third parallel branch is a 3 × 3 convolution branch, used to model local cross-channel interactions and enhance multi-scale spatial structural information, which is particularly beneficial for characterizing the elongated snake-like channels and irregular defect forms. Finally, the module synthesizes the context information from the two scales of 1 × 1 and 3 × 3 to learn the attention weights in the spatial dimension, which are used to weight the intra-group features; a Sigmoid function is then applied to the weighted results, followed by element-wise multiplication with the group features to obtain the output feature map. This suppresses noise responses caused by reflections, brightness fluctuations, and repetitive textures in complex backgrounds, enhancing the expression stability of defect discriminative features. The detailed process is shown in the figure.

**Figure 8:**
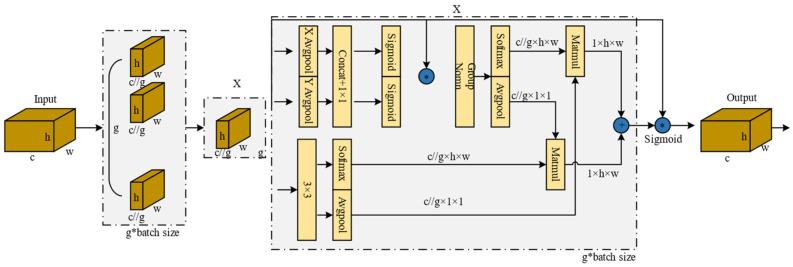
Network architecture diagram of the Efficient Multi-scale Attention (EMA) mechanism.

**Figure 9:**
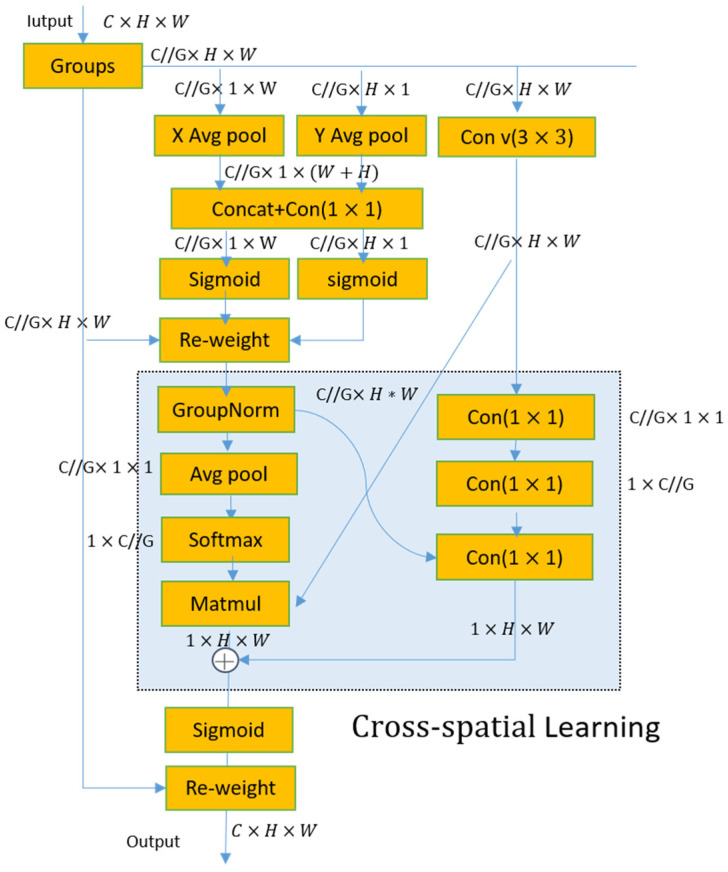
The structure of the EMA module based on grouping strategy.

As shown in Figure 8, the EMA module effectively reduces computational overhead and achieves multi-scale feature extraction by grouping input features and employing multi-branch processing. However, the core of this module resides in the realization of information interaction across spatial dimensions. To this end, Figure 9 provides a detailed illustration of its internal “cross-spatial learning” computational logic, which performs aggregation and adaptive recalibration of the outputs from the 1 × 1 and 3 × 3 branches to ultimately achieve high-quality feature fusion.

#### 2.2.4. Dynamic Snake Convolution

Instead of simply reducing redundancy as the standard SCConv does, our approach gives the convolutional kernel the ability to physically adapt. Relying on a cumulative offset mechanism, the kernel dynamically deforms to perfectly match the winding, snake-like layout of the channels. This built-in flexibility makes DySnakeConv much more effective at extracting features from slender and irregularly shaped defects. As a result, the network’s overall performance sees a significant boost when dealing with these strict geometric constraints. The specific structural breakdown is detailed below.

**Figure 10:**
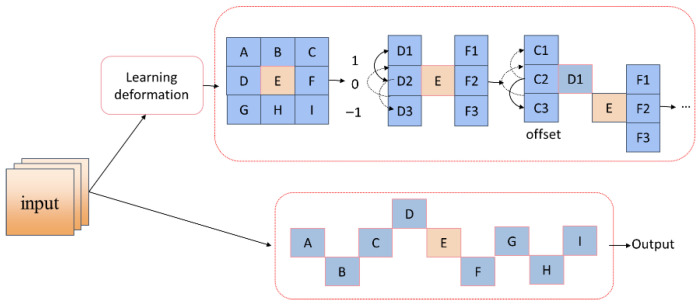
Schematic illustration of the Dynamic Snake Convolution operation.

Figure 10 highlights exactly how the DySnakeConv deforms in practice. Instead of sticking to a rigid grid, every element inside the standard kernel (such as point E and its immediate neighbors) learns to generate cumulative offsets by analyzing the local shapes of the input features. By breaking away from conventional square sampling, the kernel gains the flexibility to stretch and bend along the labyrinth flow channels, much like a snake. Ultimately, this structural freedom makes the network far more effective at distinguishing features under strict geometric constraints.

We upgraded the baseline YOLOv8 bounding box loss from CIoU to Inner-SIoU. The original CIoU often struggles with adaptive adjustments and overall generalization across diverse scenarios. By integrating the Inner-SIoU framework [30], we significantly speed up the Bounding Box Regression (BBR) convergence while making the model much more robust. At its core, this BBR process relies on the fundamental IoU calculation detailed in Formula (5).

(5) $$IoU=\frac{\left|B\cap B^{gt}\right|}{\left|B\cup B^{gt}\right|}$$

**Figure 11:**
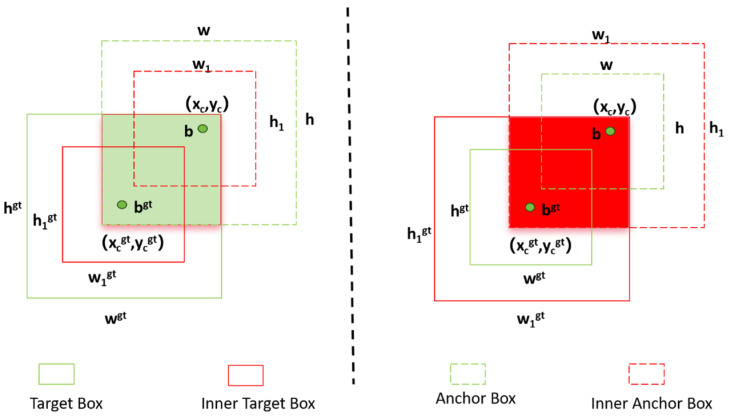
Inner-IoU structure.

To ensure that the model can capture more spatial features in the image, the overlap between the ground truth box $B^{gt}$ and the prediction box B is calculated. Building upon this, the SIoU loss function introduces an angle loss Λ to consider the impact of the angle between the anchor box and the truth box $B^{gt}$. Combined with the distance loss Δ and shape loss Ω defined in Equations (6)–(8), it refines the regression logic as shown in Figure 11.

(6) $$L_{SIoU}=1-IoU+\frac{\left(\Delta +\Omega \right)}{2}$$

(7) $$\Delta =\frac{1}{2}\sum_{t=w,h}\left(1-e^{-\gamma \rho t}\right),\gamma =2-\Lambda$$

(8) $$\Omega =\frac{1}{2}\sum_{t=w,h}{\left(1-e^{w_{t}}\right)}^{\theta },\theta =4$$

This method achieves key innovation by using a novel auxiliary bounding box mechanism, which effectively controls the dimension proportions of the auxiliary boxes via a scaling factor ratio typically within the range of [0.5, 1.5]. The coordinates of the auxiliary ground truth box ($b^{gt}$) and the anchor box (b) are derived by proportionally scaling the original dimensions according to Formulas (9)–(12). This mechanism enables the dynamic regulation of regression gradients: when ratio < 1, smaller auxiliary boxes generate larger absolute gradient values, accelerating the convergence of high-IoU samples; when ratio>1, the auxiliary boxes expand the effective regression range.

(9) $$b_{t}^{gt}=y_{c}^{gt}-\frac{h^{gt}\;*\;\mathrm{ratio}\;}{2},b_{b}^{gt}=y_{c}^{gt}+\frac{h^{gt}\;*\;\mathrm{ratio}\;}{2}$$

(10) $$b_{l}=x_{c}-\frac{w*\;\mathrm{ratio}\;}{2},b_{r}=x_{c}+\frac{w*\;\mathrm{ratio}\;}{2}$$

(11) $$b_{t}=y_{c}-\frac{h*\;\mathrm{ratio}\;}{2},b_{b}=y_{c}+\frac{h*\;\mathrm{ratio}\;}{2}$$

(12) $$inter=(min(b_{r}^{gt},b_{r})-max(b_{l}^{gt},b_{l}))*(min(b_{b}^{gt},b_{b})-max(b_{t}^{gt},b_{t}))$$

(13) $$\mathrm{union}\;=(w^{gt}*h^{gt})*(\mathrm{ratio}{)}^{2}+(w*h)*(\mathrm{ratio}{)}^{2}-\;\mathrm{inter}\;$$

Finally, the complete Inner-SIoU loss function is formed by integrating the SIoU framework with auxiliary IoU components via Formula (14).

(14) $$L_{\mathrm{Inner-SIoU}\;}=L_{\mathrm{SIoU}\;}+\;\mathrm{IoU}\;-\frac{\;\mathrm{inter}\;}{\mathrm{union}\;}$$

The angle, distance, and shape losses constrain the directional deviation, center distance, and shape difference between the predicted box and the ground-truth box, respectively, thereby improving localization stability for small-scale and irregular defects. In addition, the auxiliary bounding-box mechanism provides a more flexible optimization reference at different training stages, which helps improve regression convergence and localization accuracy.

### 2.3. Evaluation Index and Experimental Environment

To comprehensively evaluate the performance of the proposed model, this study adopts several key metrics widely recognized in the field of object detection, including Precision (P), Recall (R), Average Precision (AP), and mean Average Precision (mAP). The specific mathematical formulas are shown in Formulas (15)–(18). Among them, $T_{P}$ is the number of correctly identified positive samples, $F_{P}$ is the number of incorrectly identified positive samples, and $F_{N}$ is the number of samples that were not detected.

(15) $$P=\frac{T_{P}}{T_{P}+F_{P}}$$

(16) $$R=\frac{T_{P}}{T_{P}+F_{N}}$$

(17) $$AP=\int_{0}^{1}P(R)dR$$

(18) $$mAP=\frac{\sum_{i=1}^{n}AP_{i}}{n}$$

All model training and evaluations were conducted on a local workstation running Windows 11. The hardware configuration included an Intel Core i9-10980XE CPU, 64 GB of RAM, and an NVIDIA GeForce RTX 3090 GPU. For the software environment, we wrote the scripts in Python 3.8.19 using the PyCharm 2024 IDE. Additionally, the deep learning architectures were implemented in PyTorch 2.2.1, relying on CUDA 12.2 to handle GPU acceleration.

## 3. Results

### 3.1. Training Result

After 600 epochs of training, the improved YOLOv8 model achieved key performance metrics with (R) 90.9%, (P) of 89.6%, and mAP50 of 93.9%. The detailed identification and detection results for the six defect types—atrophied spout, irregularity, defect, contaminated, deformation, and spout blockage—are presented in Table 2. The results indicate that the proposed model exhibits strong generalization capability and robustness in the identification and detection of various defects in labyrinth emitter. Among all categories, the contaminated class attains the highest classification precision of 96.4%, and it also achieves the highest recall of 92.1%, suggesting that the model has a strong discriminative ability for surface-contamination defects and a lower risk of missed detections. The highest mAP50 of 97.3% is observed in the contaminated category, whereas the lowest mAP50 (83.7%) occurs in the detection of the deformation defect, indicating that deformation-related defects are more challenging due to their continuous appearance variations and unstable boundaries. Although Table 2 validates the effectiveness of the improved model across different defect categories, it remains necessary to quantify which proposed modules are responsible for the overall performance gains. Therefore, an ablation study is conducted by progressively enabling each component and comparing metric changes to clarify both individual contributions and synergistic effects, as reported in Table 3.

### 3.2. Ablation Study

To systematically evaluate the contribution of each proposed module to the performance improvement of the YOLOv8 detector, an ablation study was conducted. Different optimization strategies were progressively introduced, and their impacts on detection performance were compared and analyzed. The experimental results are summarized in Table 3. The results show that after incorporating the DySample dynamic upsampling module, the model’s recall increased by approximately 2.3%. The defect feature details of labyrinth emitter are better preserved during the feature fusion stage of this model, thereby effectively reducing missed detections. After further integrating the EMA mechanism into the backbone network, the recall and mAP50 improved by 2.3% and 0.2%, respectively, suggesting that multi-scale attention modeling enhances the response to key regions and improves the discriminative capability of feature representations. Subsequently, the introduction of the DySnakeConv module in the feature fusion and propagation stages led to a 2.8% increase in precision, demonstrating that this structure is more adaptive for modeling irregular and curved targets and can effectively suppress false detections.

On this basis, the original bounding-box regression loss was replaced with Inner-SIoU, which strengthens the optimization constraints on internal overlap regions and geometric consistency, thereby further improving localization accuracy. With all the above strategies combined, compared with the baseline object detection model YOLOv8, the proposed method achieves improvements of 7.3%, 3.9%, and 3.3% in the key performance metrics of precision (P), recall (R), and mAP50, respectively., recall, and mAP50, respectively, compared with the baseline, fully demonstrating the synergistic effects and cumulative gains brought by the proposed modules.

**Figure 12:**
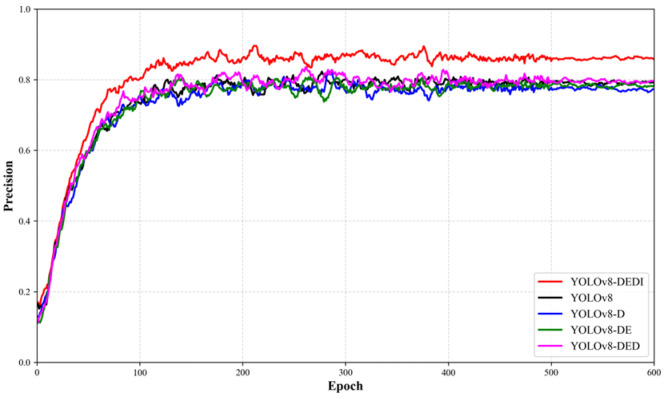
Variation curves of Precision for different YOLOv8 improvements during the training process.

**Figure 13:**
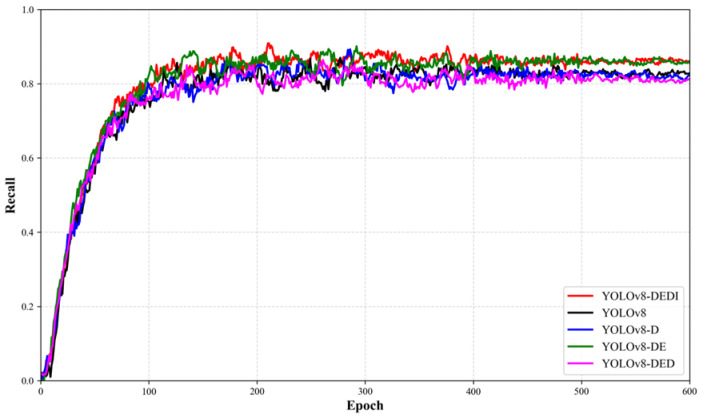
Variation curves of Recall for different YOLOv8 improvements during the training process.

**Figure 14:**
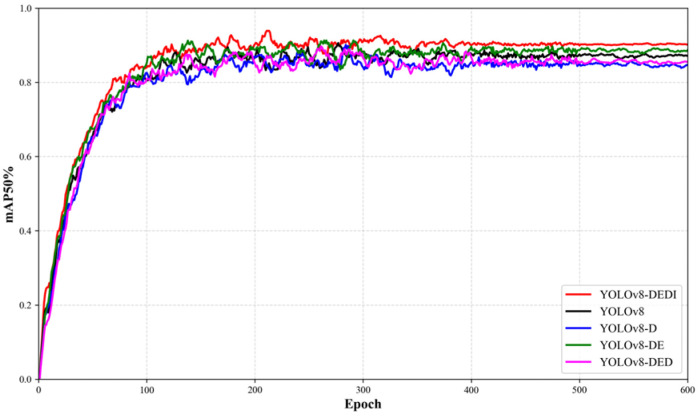
Variation curves of mAP50%for different YOLOv8 improvements during the training process.

Beyond the comparison of final metrics, different module configurations may also affect convergence speed and training stability. To visualize the training dynamics, the curves of Precision, Recall, and mAP50 versus epochs are further plotted for each model variant to analyze convergence behavior and stability differences, as shown in Figure 12, Figure 13 and Figure 14.

**Figure 15:**
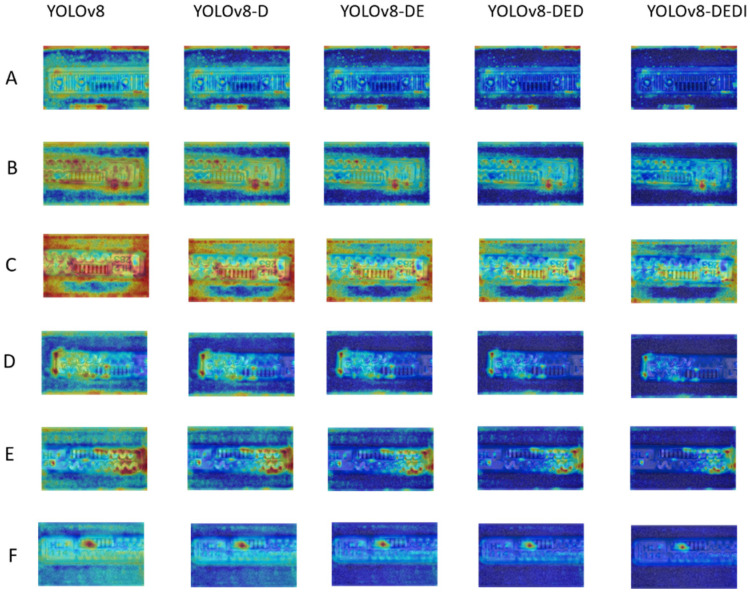
Different improvements of YOLOv8 in visual thermal map effect presentation ((**A**) AtrophiedSpout; (**B**) Irregularity; (**C**) Defect; (**D**) Contaminated; (**E**) Deformation; (**F**) SpoutBlockage).

To comprehensively evaluate the interpretability and attention mechanism of the improved model in the task of Labyrinth Emitter defect detection, this study employs Gradient-weighted Class Activation Mapping (Grad-CAM) to perform a visual analysis of the key focus areas during the model’s prediction process. Grad-CAM can highlight the discriminative regions that the model relies on when making classification and localization decisions, thereby helping to clarify the basis for the model’s predictions [31]. As shown in Figure 15, this paper compares the attention heatmap performance of various YOLOv8 improvements across six defect categories. The color intensity in the heatmaps reflects the model’s degree of focus on specific areas, with darker colors indicating closer attention. The results demonstrate that, although some false positives and false negatives still exist, the baseline YOLOv8 is already capable of focusing on the primary discriminative regions of the Labyrinth Emitter defects. After introducing DySample, the model’s attention is refined, concentrating more on defect edges and the structural details of the flow channels. Further integration of EMA leads to a more uniform and stable attention distribution. Building upon this, the addition of DySnakeConv strengthens the response to slender, curved channels and key defect areas. Ultimately, the fully improved YOLOv8 exhibits deeper and more concentrated response regions in the target prediction heatmaps, indicating a significant enhancement in its focus on the areas of interest within the target. Combined with the optimization constraints of Inner-SIoU, the attention allocation becomes even more rational, allowing for more accurate highlighting of the most discriminative defect regions in the Labyrinth emitter.

### 3.3. Comparison of Different Target Detection Models

**Figure 16:**
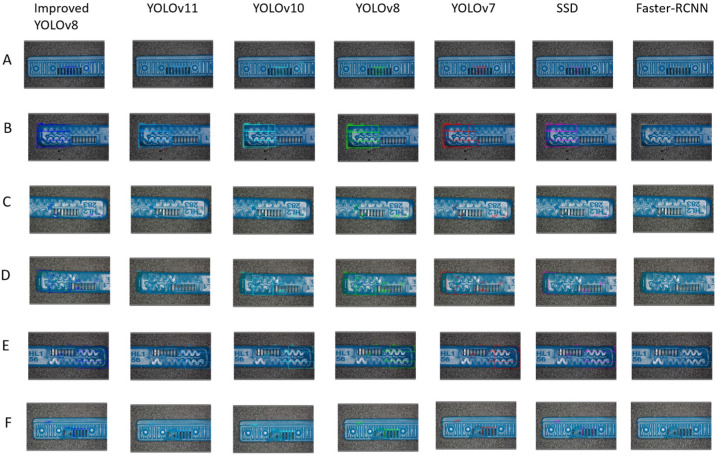
Identification results of different models (**A**) AtrophiedSpout; (**B**) Irregularity; (**C**) Defect; (**D**) Contaminated; (**E**) Deformation; (**F**) SpoutBlockage.

From the perspective of model complexity and inference speed (as shown in Table 4), the traditional two-stage detector Faster R-CNN shows clear limitations for real-time industrial inspection, with 41 M parameters and only 11 FPS. In contrast, single-stage detectors, especially the YOLO series, provide substantially higher inference efficiency. The improved YOLOv8 proposed in this study achieves 89.6% precision, 90.9% recall, and 93.9% mAP50, which are higher than those of YOLOv8, YOLOv10, and YOLOv11 in accuracy-related metrics on the constructed dataset. Although its absolute inference speed (150.2 FPS) is slightly lower than that of YOLOv10, YOLOv11, and the baseline YOLOv8, it still maintains competitive real-time performance together with a relatively lightweight model size of 3.7 M parameters. Therefore, the results in Table 4 indicate that the proposed model improves detection accuracy while preserving competitive efficiency, rather than demonstrating a universal speed advantage. Combined with the visual results in Figure 16, the proposed model shows better overall detection effectiveness for the defect categories considered in this study.

In summary, traditional models such as SSD and Faster R-CNN show clear disadvantages in either accuracy or real-time performance for the present task, while YOLOv8-series models provide a more suitable basis for industrial deployment. Compared with YOLOv10 and YOLOv11, the improved YOLOv8 model in this study does not exhibit the highest FPS, but it achieves better precision, recall, and mAP50 on the constructed dataset while still maintaining competitive real-time performance and a relatively lightweight model size. Therefore, its advantage is more appropriately reflected in a favorable accuracy–efficiency trade-off under the current experimental setting.

To verify that the performance improvement of Improved YOLOv8 over the baseline YOLOv8 was not caused by random variation, two statistical tests were introduced in this study. First, a Bootstrap resampling method was used to estimate the 95% confidence interval (CI) of the mAP50 difference between the two models, so as to quantify the reliability of the observed performance gain. The mAP50 of Improved YOLOv8 was 93.90%, whereas that of the baseline YOLOv8 was 90.60%, corresponding to an average improvement of +3.30%. The results showed that the 95% CI for the mAP50 improvement of Improved YOLOv8 was [+1.10%, +5.03%]. Since the entire interval lies above zero, this indicates that the improved model achieves a stable performance gain over the baseline model. McNemar’s test was conducted to statistically analyze the paired image-level decision outcomes of the two models on the same test images. The results showed that, among the samples misclassified by the baseline model, Improved YOLOv8 corrected 22 errors while introducing only 9 new errors. The resulting *p*-value was 0.0311, which is below the significance level of 0.05. Overall, as shown in Table 5, these statistical results indicate that the performance improvement of Improved YOLOv8 over the baseline model is statistically significant rather than an accidental experimental outcome.

### 3.4. Generalization Experiment

To further evaluate the applicability of the proposed method in real production scenarios, an additional generalization experiment was conducted on the basis of the original experiments, focusing on cross-batch and cross-material-condition validation. Different from the training set, the additional validation data used in this section, as shown in Table 6, consist of original product images collected from other production batches and cover different material conditions, with a total of 350 images. All samples were manually screened by on-site quality inspectors and relevant domain experts, and were divided at the image level into seven categories, including 50 normal samples and 50 samples for each of the other six defect categories. This supplementary validation set was not involved in model training and was used only to assess the detection stability and generalization capability of the trained model under new batches and new material conditions.

**Figure 17:**
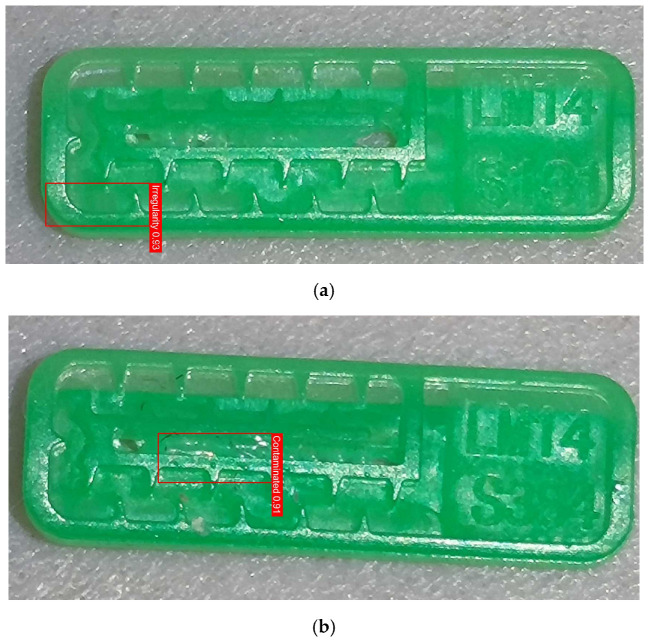
Examples of false detections on defect-free samples. (**a**) a defect-free sample incorrectly detected as Irregularity; (**b**) a defect-free sample incorrectly detected as Contaminated.

As shown in Table 7, the proposed method achieved favorable image-level recognition performance on 350 supplementary validation images collected from production batches different from those used for training and under varying material conditions, with an overall accuracy of 89.14%. Among them, the recognition accuracy for normal samples reached 96.00%, corresponding to an image-level false-positive rate of 4.00%, as illustrated in Figure 17, which indicates that the model has a relatively low false alarm risk under real deployment conditions. For the defect categories, Contaminated and SpoutBlockage showed relatively better recognition performance, whereas some confusion was observed among Irregularity, Defect, and Deformation, suggesting that defects with complex boundaries still present certain challenges for recognition under cross-batch conditions.

The experimental results indicate that the proposed method still maintains a favorable detection performance on the cross-domain dataset, demonstrating that the model has a certain degree of generalization ability under different production batches and material conditions. At the same time, the false-positive level on normal samples remains low, suggesting that the model exhibits a certain degree of stability under real deployment conditions.

### 3.5. Spatial Simulation and Virtual Prototyping for Physical Deployment

The effective operation of a digital twin relies on continuous, real-time, and controllable data streams. To establish this dynamic foundation, a high-fidelity 3D simulation environment is built to verify the consistency and accuracy of virtual–physical mapping. In the digital space, the vision acquisition hardware and key production stages are modeled in a parametric manner and organized into a reproducible offline validation platform. Based on this virtual system (Figure 4 and Figure 5), equipment layouts can be checked before deployment. Potential issues under high-speed conveying, such as spatial interference, occluded views, and installation constraints, can also be identified in advance.

The offline simulation further determines the feasible installation of the Gigabit Ethernet camera (Hikrobot MV-CA060-11GC hikvision, Hangzhou, China) and the ring light. Results show that a camera-to-target distance of approximately 0.8 m provides effective coverage of a field of view of about 0.8 m × 0.5 m while avoiding collisions with the surrounding machinery. To avoid overstating the role of the digital twin, its contribution in this study is quantified mainly from the perspective of geometric feasibility and deployment-oriented verification, rather than direct improvement of detection accuracy.

To quantify the engineering role of the digital twin platform in inspection-station layout and imaging-parameter verification, four geometric indicators, namely field-of-view dimensions, field-of-view utilization, spatial resolution, and minimum installation clearance, are introduced, as given in Equations (19)–(22).

(19) $$W_{\mathrm{FOV}}=\frac{dS_{w}}{f},H_{\mathrm{FOV}}=\frac{dS_{h}}{f}$$

(20) $$\eta_{\mathrm{FOV}}=\frac{A_{\mathrm{ROI}}}{A_{\mathrm{FOV}}}=\frac{W_{\mathrm{ROI}}H_{\mathrm{ROI}}}{W_{\mathrm{FOV}}H_{\mathrm{FOV}}}$$

(21) $$r_{x}=\frac{W_{\mathrm{FOV}}}{N_{x}},r_{y}=\frac{H_{\mathrm{FOV}}}{N_{y}}$$

(22) $$C_{min}=\underset{i,j}{min}dist(B_{i},B_{j})$$

Specifically, $W_{\mathrm{FOV}}$ and $H_{\mathrm{FOV}}$ denote the field-of-view dimensions on the object plane and are jointly determined by the working distance d, the sensor dimensions $S_{w}$ and $S_{h}$, and the lens focal length f; $\eta_{\mathrm{FOV}}$ is used to characterize the proportion of the target inspection area within the total field of view; $r_{x}$ and $r_{y}$ represent the spatial resolution of the imaging system for resolving micro-defects; and $C_{min}$ is used to measure the collision-free installation clearance of the inspection workstation. Together, these indicators provide a quantitative basis for feasibility verification in the digital twin environment. Combined with simulation analysis and on-site calibration results, the camera-to-target distance was determined to be approximately 0.8 m. Under this condition, the effective coverage of a single image is about 0.8 m × 0.5 m, which meets the overall imaging requirements of the patch conveying region while ensuring feasible installation space.

## 4. Discussion

To address the problem of online inspection of labyrinth emitter patches on active production lines, this study constructs a visual defect detection framework supported by Digital Twin (DT) technology. Focusing on key manufacturing links such as patch conveyance, station alignment, and image acquisition, a high-fidelity digital twin simulation system is established, which provides a reliable basis for acquisition-system design and offline verification before actual industrial deployment. On this basis, the study targets six typical defect categories of labyrinth emitter patches and extracts discriminative visual features to achieve accurate identification of defective products, thereby improving quality control in subsequent production processes.

To further enhance detection performance, the original YOLOv8 baseline is systematically improved from four aspects: feature reconstruction, attention enhancement, geometric adaptation, and bounding-box regression. First, DySample is introduced as a dynamic upsampling operator, which alleviates feature blurring caused by conventional interpolation-based upsampling by generating content-aware spatial offsets. Second, the Efficient Multi-Scale Attention (EMA) module is embedded to strengthen the interaction between spatial and channel information, enabling the network to focus more effectively on true defect regions while suppressing background interference. Third, in view of the slender, curved, and irregular structural characteristics of labyrinth channels, Dynamic Snake Convolution (DySnakeConv) is introduced to enhance the model’s ability to represent complex defect morphologies, especially for local blockage and boundary deformation. Finally, Inner-SIoU is used to replace the conventional bounding-box regression loss, together with an auxiliary bounding-box mechanism, so as to accelerate model convergence and improve localization accuracy for small and irregular defects. Experimental results show that the above improvements are clearly effective. The final model achieves 89.6% precision, 90.9% recall, and 93.9% mAP@0.5, representing improvements of 7.3%, 3.9%, and 3.3%, respectively, over the original YOLOv8 baseline. Compared with mainstream detectors such as YOLOv11, YOLOv10, YOLOv7, SSD, and Faster R-CNN, the proposed method shows strong overall competitiveness while still maintaining a high inference speed of 150.2 FPS, indicating its potential for real-time deployment in industrial production environments.

In addition, although the proposed method is designed for industrial inspection scenarios, the current validation is still mainly conducted under a fixed inspection-station setting and controlled acquisition conditions. Systematic robustness evaluation under more challenging perturbations, such as motion blur, illumination fluctuation, reflection, slight vibration, defocus, and partial occlusion, has not yet been carried out. Future work will further expand the dataset and testing protocol to validate the method under more complex industrial disturbances, while deepening the application of digital twin technology in extreme-scenario construction to improve environmental adaptability and better support industrial edge-computing deployment.

## 5. Conclusions

Detecting micro-defects in labyrinth emitter patches on high-speed production lines remains a key challenge in drip-irrigation product manufacturing. Conventional machine-vision systems often find it difficult to balance detection accuracy and processing speed, which can easily lead to false alarms and missed detections under continuous industrial operation. To address this issue, this study proposes a Digital Twin (DT)-assisted inspection framework for production lines. As an offline verification platform, the DT system can simulate key manufacturing stages, such as patch conveying, station alignment, and image acquisition, before on-site deployment, thereby supporting structural layout validation and acquisition-system optimization. Meanwhile, the defect detection model itself is trained and tested using real images collected from a physical inspection platform. This virtual-real collaborative workflow reduces engineering trial-and-error and improves the feasibility of subsequent industrial deployment.

On this basis, this study develops a highly sensitive and lightweight defect detection model through a systematic improvement of the YOLOv8 architecture. To better capture the fine structures and irregular boundaries of labyrinth channels, the model is enhanced from four aspects: dynamic feature reconstruction, attention-guided representation, geometric adaptation, and regression optimization. First, DySample is introduced to preserve finer spatial details during feature upsampling. Second, an EMA module is embedded to enhance feature discrimination and suppress background interference. Third, in view of the slender, curved, and tortuous morphological characteristics of labyrinth regions, DySnakeConv is incorporated to improve the model’s adaptive perception of complex defect boundaries. Finally, Inner-SIoU is adopted to optimize bounding-box regression and accelerate model convergence during training.

Experimental results on six typical defect categories show that the proposed method performs well. The final model achieves 89.6% precision, 90.9% recall, and 93.9% mAP@0.5, while containing only 3.7 M parameters and maintaining an inference speed of 150.2 FPS. In addition to the standard test results, a supplementary generalization experiment is conducted under practical production conditions. An additional image-level validation set containing 350 samples is constructed. These samples are collected from different production batches and different material conditions and are independent of the training data, so as to further evaluate the model’s cross-batch robustness. The proposed method achieves an overall image-level accuracy of 89.14% on this supplementary dataset. In particular, the recognition accuracy for normal samples reaches 96.00%, corresponding to a false-positive rate of only 4.00%, indicating that the method has a relatively low false-alarm risk under real deployment conditions. These results show that the proposed method not only performs well on the original test set but also maintains good applicability when batch and material conditions change.

## Figures and Tables

**Table 1:** Defect Type Dataset.

Defect Type	Defect Sample	Label Name	Number of Images
AtrophiedSpout	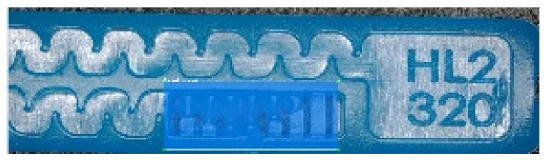	AtrophiedSpout	500
Irregularity	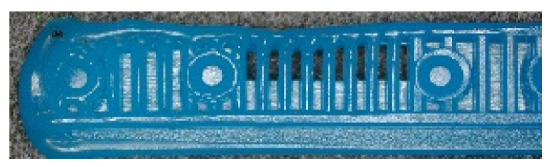	Irregularity	500
Defect	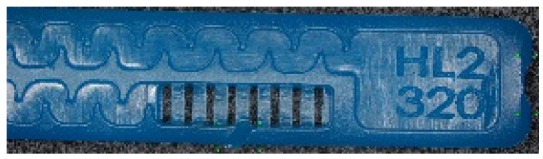	Defect	500
Contaminated	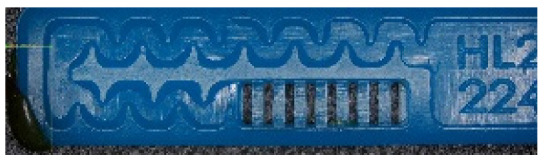	Contaminated	500
Deformation	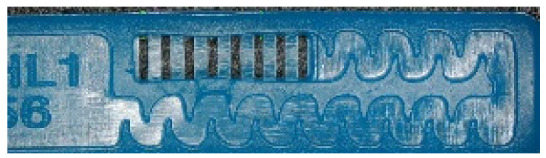	Deformation	500
SpoutBlockage	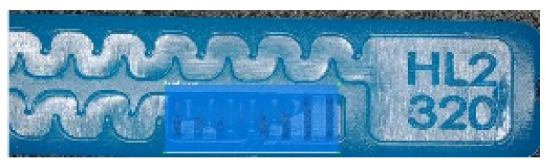	SpoutBlockage	500
Total			3000

**Table 2:** Training results of the improved YOLOv8 model.

Defect Type	P%	R%	mAP50%
AtrophiedSpout	88.3	81.5	86.4
Irregularity	92.3	91.5	96.3
Defect	93.7	89.9	93.1
Contaminated	96.4	92.1	97.3
Deformation	87.4	80.1	83.7
SpoutBlockage	84.6	90.6	92.9

**Table 3:** Comparison of ablation performance.

	Model				P%	R%	mAP50%	Parameters/M	FPS
Yolov8	Dysample	EMA	DSConv	Inner-SIoU	-	-	-	-	-
	✗	✗	✗	✗	82.3	87.0	90.6	3.1	165.4
	✔	✗	✗	✗	82.3	89.3	90.1	3.3	78.2
	✔	✔	✗	✗	80.7	90.1	91.3	3.7	71.7
	✔	✔	✔	✗	83.5	86.4	89.7	3.9	59.3
	✔	✔	✔	✔	89.6	90.9	93.9	3.7	150.2

✗ means without this module, and ✔ means with this module.

**Table 4:** Comparison of experimental results of different network models.

Model	P%	R%	mAP50%	Parameters/M	FPS
ImprovedYolov8	89.6	90.9	93.9	3.7	150.2
YOLOv11	84.8	85.9	91.3	2.8	159.9
YOLOv10	79.6	83.2	86.6	2.7	179
YOLOv8	82.3	87.0	90.6	3.1	167
YOLOv7	79.1	75.3	81.0	9.3	66
SSD	75.1	73.8	79.6	32	51
Faster-RCNN	76.4	76.9	80.2	41	11

**Table 5:** Statistical tests of the performance of Improved YOLOv8 relative to the baseline model.

Statistical Test	Metric	Result	Conclusion
Bootstrap Test	95% CI for mAP50 Difference	[+1.10%, +5.03%]	Significant (Interval does not contain 0)
McNemar’s Test	p-value	p = 0.0311 (<0.05)	Significant (p < 0.05)

**Table 6:** Generalization Experiment Dataset.

Defect Type	Defect Sample	Label Name	Number of Images
AtrophiedSpout	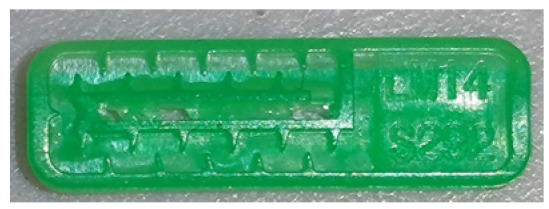	AtrophiedSpout	50
Irregularity	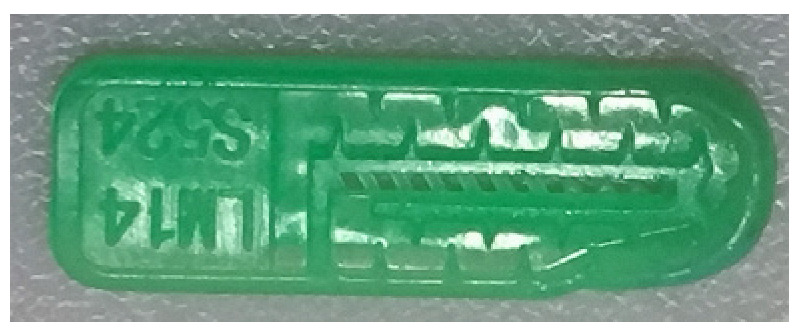	Irregularity	50
Defect	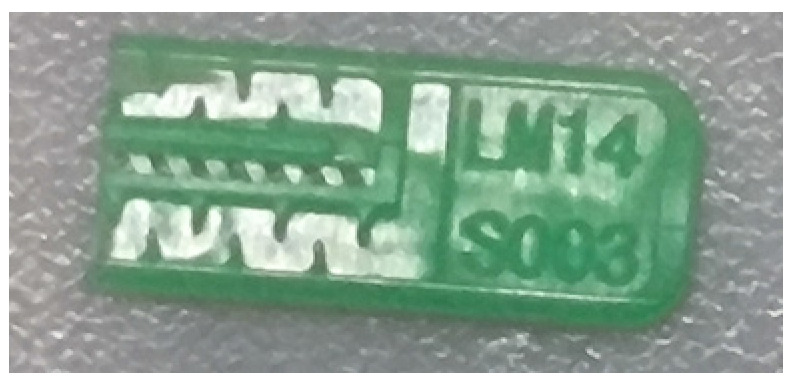	Defect	50
Contaminated	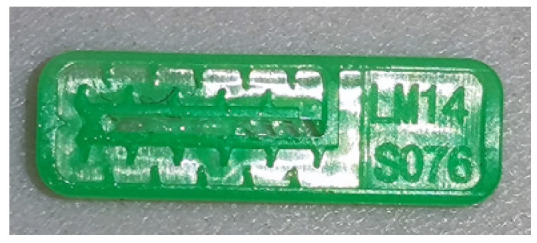	Contaminated	50
Deformation	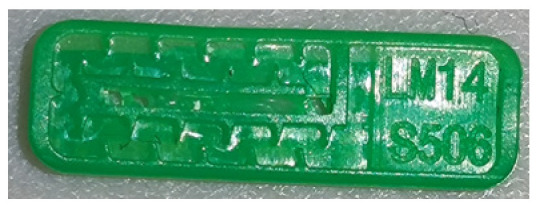	Deformation	50
SpoutBlockage	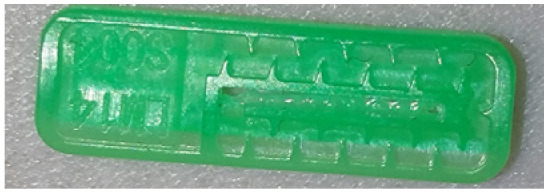	SpoutBlockage	50
Normal	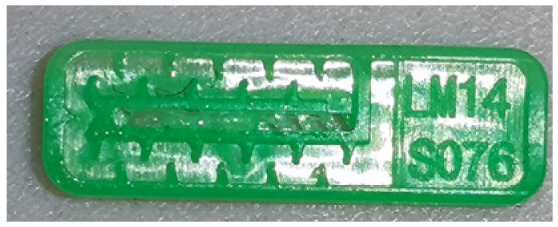	Normal	50
Total			350

**Table 7:** Classification Results of Different Categories in the Generalization Experiment.

Class	Number of Samples	Correctly Identified Samples	Accuracy %
AtrophiedSpout	50	45	90.00
Irregularity	50	42	84.00
Defect	50	41	82.00
Contaminated	50	46	92.00
Deformation	50	43	86.00
SpoutBlockage	50	47	94.00
Normal	50	48	96.00
Overall	350	312	89.14

## Data Availability

The data presented in this study are available on request from the corresponding author due to confidentiality restrictions related to industrial production and commercial interests.
